# Undifferentiated liver sarcoma – rare entity: a case report and review of the literature

**DOI:** 10.1186/1752-1947-2-20

**Published:** 2008-01-25

**Authors:** Kashif Iqbal, Zhang Meng Xian, Chen Yuan

**Affiliations:** 1 Nuclear Medicine, Oncology and Radiotherapy Institute, G-8/3, Islamabad, Pakistan; 2 Cancer Center, Tongji Hospital, Tongji Medical College. Huazhong University of Science and Technology. Wuhan. China

## Abstract

**Introduction:**

Undifferentiated Liver Sarcoma, also known as Undifferentiated Embryonal Sarcoma of the Liver, is a rare, highly malignant neoplasm which affects mostly the pediatric population, although a few cases have been reported in adults. It accounts for about 13% of pediatric hepatic malignancies.

**Case presentation:**

We report a case of undifferentiated liver sarcoma in a 14-year-old Chinese boy who presented with non-specific right hypochondriac pain. Exploratory laparotomy with tumor resection was performed, followed by adjuvant chemotherapy.

**Conclusion:**

Undifferentiated Liver Sarcoma is a rare, highly malignant hepatic neoplasm affecting almost exclusively the pediatric population. The prognosis is poor but recent evidence shows that long-term survival is possible after complete surgical resection and postoperative chemotherapy.

## Introduction

Undifferentiated Liver Sarcoma (ULS), also known as Undifferentiated Embryonal Sarcoma of the Liver, is a rare, highly malignant neoplasm which affects mostly the pediatric population, although a few cases have been reported in adults. It accounts for about 13% of pediatric hepatic malignancies. Only about 150 cases have been reported in the literature. Undifferentiated Liver Sarcoma was named as a separate entity by Stocker et al on the basis of an Armed Forces of Pathology (AFIP) series. [1] The prognosis is poor but recent evidence indicates that modern surgical procedures along with neo adjuvant or adjuvant chemotherapy have led to an increase in the median survival. We report a case of a 14 year old Chinese boy with Undifferentiated Liver Sarcoma.

## Case presentation

**Figure 1:**
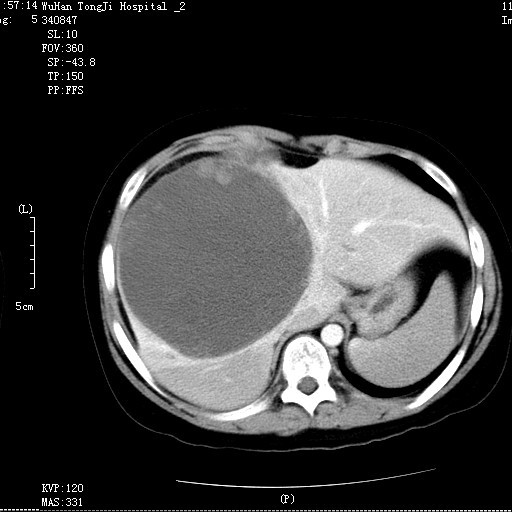
Contrast enhanced CT scan showing hepatomegaly and a large hypodense lesion in the liver.

**Figure 2:**
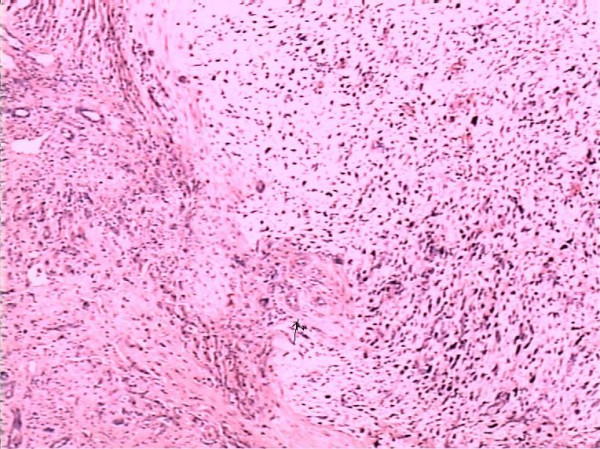
Microphotograph showing malignant sarcomatous tissue with giant neoplastic cells (H.E Staining × 100).

A 14 year old Chinese boy presented to a district hospital with a 5 day history of non-specific right hypochondriac pain. There was no history of jaundice, fever, anorexia or weight loss. He did not have any other associated symptoms. He had no significant past or family history, and no history of drug intake or allergies. His general physical examination was unremarkable. Yellowish discoloration of skin or sclera, spider naevi and palmar erythema all were absent. Systemic examination revealed massive hepatomegaly. His blood count and liver function tests were normal. Alpha fetoprotein was also normal. Ultrasonography revealed a large mass in the right lobe of the liver. He was referred to our hospital for further management. Contrast enhanced CT Scan revealed a large, hypodense mass of 14 × 15 × 15 cm in the right lobe of the liver. (Figure 1). Exploratory laparotomy was performed and revealed a large mass in the right lobe of the liver and part of the left lobe with ruptured capsule and the ruptured part adhered to pleura. Tumor resection was performed and about 70% of the total liver was resected. Pathologic review of the specimen revealed an 1150 gm right hepatic lobe and part of the left hepatic lobe with a 14 × 15 × 15 cm tumor mass. The histological examination showed malignant sarcomatous tissue with giant neoplastic cells and residual hepatocytes suggestive of Undifferentiated Liver Sarcoma. (Figure 2). Sarcomatous tissue with severe atypia of the neoplastic cells and focal presence of giant cells, partially with myoblastic characteristics, was also present. Immunohistochemical staining was positive for Phosphoenolpyruvate Carboxy Kinase (PCK), Vimentin and Alpha 1 Antitrypsin and negative for Epithelial Membrane Antigen (EMA).

Our patient's postoperative recovery was uneventful. He was given 6 cycles of adjuvant chemotherapy, AIM Regimen (Adriamycin, Ifosfamide and Mesna), which was well tolerated. A post-chemotherapy CT scan showed no signs of recurrence and our patient is alive and well 6 months after surgery.

## Discussion

Undifferentiated Liver Sarcoma is a rare, highly malignant neoplasm almost exclusively found in the pediatric population although a few cases have been reported in adults. Undifferentiated Liver Sarcoma was described as a separate pathological entity by Stocker et al in 1978. [1] Before that time different nomenclature was used, most commonly Malignant Mesenchymoma, although Primary Sarcoma of the Liver, Fibromyxosarcoma and Mesenchymoma were also used. [2].

Primary hepatic neoplasms are the third most common solid malignant tumors in children after Wilms Tumour and Neuroblastoma and they account for about 2% of total solid pediatric malignancies. Primitive mesenchymal tumor, although rare, is the fourth most common pediatric malignant hepatic tumor following Hepatoblastoma, Infantile Haemangioendothelioma and Hepatocellular Carcinoma. Primitive mesenchymal tumors represent about 9%-15% of all hepatic tumors in children. Only about 150 cases have been reported in the literature.

ULS is a malignancy of older children with the majority of cases diagnosed in children between the ages of 6–10 years. The incidence decreases after 10 years of age. There is a slight male preponderance in children (1:0.65). [2] The right lobe of the liver is more commonly involved.

Stocker et al presented a series of 31 cases which showed ULS as having a poor prognosis with median survival of less than a year. Recent evidence shows that, with the advent of modern surgical procedures and the use of pre and post operative chemotherapy, there has been an increase in the overall survival rate. A recent review of published cases revealed a better outlook with 37.5% of patients alive without disease for an average of 37.5 months.[3] Two further patients were reported alive and free of disease more than 5 years after complete surgical excision of the tumor and chemotherapy.[3]

The clinical features of ULS are often non-specific and may have varied presentation ranging from sharp abdominal pain, fever, anorexia, diarrhea or a solitary liver cyst. In our case the only presenting complaint was non-specific right hypochondriac pain. Fever is usually due to hemorrhage and necrosis. Rupture into the tumor, peritoneal cavity or pleura can be present. [4] Jaundice is usually absent. In contrast with Primary Liver Carcinoma, ULS has no relationship with hepatitis or cirrhosis. There is usually no abnormality in liver function and a normal alpha fetoprotein. Laboratory studies are usually non-specific. [1,2]

Radiographs of the abdomen are usually normal. The lesion can be detected by ultrasound, CT and MRI. MRI localizes the lesion more accurately than the other methods, with good resectability correlation. It also can detect vascular invasion, biliary obstruction and hilar adenopathy. [5].

ULS is a neoplasm with a primitive mesenchymal phenotype. Tumor size often exceeds 10 cm and they can be as large as 30 cm. Buetow et al reviewed the pathological and radiological findings in 28 cases of ULS. [6] Pathologically, the tumor is usually a large, solitary mass, predominantly solid, with the rest of it being cystic, filled with serosanguineous fluid. On ultrasonography, the lesion appears predominantly solid (iso-hyperechoic when compared to liver parenchyma) with a few cystic areas due to cystic degeneration or hemorrhage. On CT it appears predominantly hypodense, foci of hemorrhage appear hyperdense and occasionally a fluid-debris level is also noted. On MRI, the lesion is predominantly of CSF signal intensity with areas of cystic degeneration appearing hypointense on T1 weighted images and hyperintense on T2 weighted images; there can be hyperintense foci on T1W images due to hemorrhage. [7] Occasionally, the lesion can be predominantly cystic on ultrasonography and may be mistaken for a hydatid cyst. [8]

Although there is no standard treatment mentioned in the literature for Undifferentiated Liver Sarcoma, surgery with neo adjuvant or adjuvant chemotherapy [9] remains the option of choice. Considering the usual large size of the tumor, neo adjuvant or preoperative chemotherapy seems to be a logical and reasonable choice which may result in tumor shrinkage enabling complete resection. Recent researchers have shown that neo adjuvant or adjuvant chemotherapy, and/or radiotherapy when necessary, can remarkably improve a patient's survival. [10] As local recurrence and distant metastases are common, especially to peritoneum, pleura and lung, and rarely to the inferior vena cava, it is worth recommending that adjuvant chemotherapy (with Adriamycin, Ifosfamide and Mesna) be considered in patients presenting with this rare tumor. As there are no serum markers to evaluate the response or predict local recurrence, regular abdominal ultrasound or CT scan should be considered for evaluation and to look for any possible recurrence.

## Abbreviations

ULS: Undifferentiated Liver Sarcoma; UELS: Undifferentiated Embryonal Liver Sarcoma; PCK: Phosphoenolpyruvate Carboxy Kinase; EMA: Epithelial Membrane Antigen.

## Competing interests

The author(s) declare that they have no competing interests.

## Authors' contributions

All authors read and approved the final manuscript.

## Consent

Written consent was obtained from the patient's parents for publication of this case report and accompanying images. A copy of the written consent is available for review by the Editor-in-chief of this journal.
